# Quantum-Dot-Based Immunochromatographic Assay for Total IgE in Human Serum

**DOI:** 10.1371/journal.pone.0077485

**Published:** 2013-10-30

**Authors:** Anna N. Berlina, Nadezhda A. Taranova, Anatoly V. Zherdev, Mikhail N. Sankov, Igor V. Andreev, Alexandr I. Martynov, Boris B. Dzantiev

**Affiliations:** 1 A.N. Bach Institute of Biochemistry, Russian Academy of Sciences, Moscow, Russia; 2 Institute of Immunology, Russian Federal Medico-Biological Agency, Moscow, Russia; National Central University, Taiwan

## Abstract

To rapidly quantify total immunoglobulin E levels in human serum, we developed a novel quantum-dot-based immunochromatographic assay that employs digital recording of fluorescence. It can detect IgE levels of 5–1000 kU/L, with a coefficient of variation ranging from 2.0 to 9.5%. The assay can be processed in 10 min. The developed assay was tested on 95 serum samples. The correlation coefficient between the IgE values obtained by the proposed assay and those obtained by a commercial ELISA kit was 0.9884. Our assay thus shows promise as a new diagnostic tool for IgE detection.

## Introduction

More than a quarter of the world’s population suffers from allergic diseases such as bronchial asthma, allergic rhinitis, and dermatitis. Immunoglobulins of type E (IgE) play a major role in allergen recognition and the initiation of allergic reactions. IgE are located on the surface of mast cells and basophils; upon interactions with allergens they cause cell degranulation, leading to the release of mediators that induce allergic reactions [1], [2].

Normally, the content of IgE in human blood is less than 0.001% of the total amount of all immunoglobulins [3]. IgE concentration is typically expressed in kU/L, where 1 kU/L corresponds to 2.4 µg/L [4], [5]. The concentration of total IgE in a healthy adult is about 80 kU/L [6], [7]. In the case of allergic disease or myeloma, the IgE content in blood is increased 4–30 fold [8], [9]. Therefore, methods for determining the total IgE concentration are necessary for primary care providers to assess the state of the immune system and quickly refer patients for further examination.

Clinical diagnostic laboratories mainly use the enzyme-linked immunosorbent assay (ELISA) to determine the total IgE [10]. Available ELISA kits determine total IgE in the range of 5–1000 kU/L; however, the assay requires at least 2 hours for obtaining the results [11]–[13].

Immunochromatographic assays are attractive alternatives to ELISA because they are faster and less labor-intensive [14]. Existing commercially available tests can determine the total IgE in human serum in 7–25 minutes [15], [16]. These tests use colloidal gold as a label; its binding is detected visually or by an optical detector as the appearance of coloration in target zones of the strip. However, when working with complex matrices such as bodily fluids, the labels and sample components can cause significant unspecific staining of the test strip, hampering reliable measurements of low analyte concentrations.

Currently available immunochromatographic tests for human serum can detect total IgE with qualitative precision. For example, the ALFA Total IgE test from Dr. Fooke Laboratorien (Neuss, Germany) [16] indicates the levels of total IgE in human serum or plasma by the appearance from one to three colored lines. The quantitative determination of total IgE is, however, the most relevant for decisions to pursue further therapy [17]; this information, at present, can only be obtained via the Milenia Biotec (Giessen, Germany) MQTE 1 immunochromatographic test, based on a colloidal gold label and makes use of a portable reader with software to rapidly obtain quantitative results. However, this system has a relatively high detection limit of 30 kU/L [15].

The use of other labels, such as fluorescent markers, in immunochromatographic assay may help decrease the detection limit and decrease the influence of the matrix. Several studies have reported low detection limits for assays based on fluorescent nanoparticles [18], [19]. Quantum dots (QDs) have also been investigated as fluorescent labels for immunoassays [20]–[25]. QDs are semiconductor nanocrystals whose diameter varies from 2 to 10 nm; their fluorescence emission peak strongly depends on their size as well as their composition. Water-soluble QDs have a surface coating enriched with carboxyl or amine groups, thus facilitating their conjugation with antibodies [22], [26]. QDs, in comparison with organic fluorescent labels, are more stable, have a narrow and symmetrical emission maximum, and are resistant to photobleaching [27]. QDs thus show promise as bioanalytical labels. There are several publications describing QD-based immunodetection of human IgE by using such techniques as ELISA [28] and phosphorescent immunoassay [29]. However, the use of QDs in immunochromatographic assays has not been previously reported.

The purpose of our study was to develop an immunochromatographic test, using QDs as a label, for the determination of total IgE in human serum. Levels of IgE were assessed with a portable fluorescence detector REFLEKOM-UV, containing a UV light source [30], which is amenable to clinical work or field studies as well as laboratory conditions. The paper describes results of a technical development, including the optimization of the assay conditions and testing of the immunochromatographic assay using serum samples.

## Materials and Methods

### 1. Reagents

Water-soluble quantum dots were obtained from Invitrogen (Eugene, OR, USA). The QDs were composed of CdSe/ZnS and coated with carboxylated polymer, and their emission maximum was 625 nm. The non-ionic detergent Tween 20 and bovine serum albumin (BSA) were obtained from Sigma (St. Louis, MO, USA). Clone 4F4 of anti-human IgE monoclonal antibodies was obtained from Polygnost (St. Petersburg, Russia) and clone ME-113 was obtained from GeneTex (Irvine, CA, USA). Goat anti-mouse IgG polyclonal antibodies were obtained from IMTEK (Moscow, Russia). N-(4-dimethylaminopropyl)-N’-ethyl-carbodiimide hydrochloride (EDC) and sodium N-hydroxysulfosuccinimide (sulfo-NHS) were obtained from Fluka (St. Gallen, Switzerland). Sephadex G-100 was obtained from MP Biomedicals (Solon, OH, USA). Standard solutions of IgE with concentrations of 5, 20, 50, 100, 200 and 1000 kU/L were obtained from Dr. Fooke Laboratorien (Neuss, Germany). All other reagents were obtained from Chimmed (Moscow, Russia). Deionized water, with a resistance of 18.2 MΩ • cm at 25°C, was obtained using a Simplicity system from Millipore (Bedford, MA, USA) and used to prepare all aqueous solutions.

CNPC-backed nitrocellulose membranes and pretreated TYPE-GBF-R7L sample pads were obtained from Advanced Microdevices (MDI, Ambala Cantt, India). The following materials were purchased from Millipore (Bedford, MA, USA): CFSP223000 adsorption pads, Amicon Ultra 100 kDa centrifugal filter units, and fiberglass macroporous CFCP203000 conjugate pads for application of quantum dot conjugates.

We used the following buffers: 50 mmol/L phosphate buffer supplemented with 100 mmol/L NaCl, pH 7.4 (PBS 50), 10 mmol/L phosphate buffer supplemented with 100 mmol/L NaCl, pH 7.4 (PBS 10), and 50 mmol/L borate buffer, pH 8.5 (BB).

### 2. Collection and Storage of Biological Samples

Blood samples were collected from subjects (95 men and women, 18–60 years old) during allergy consultations as part of a routine health check-up at the Institute of Immunology (hospital department), Moscow, Russia. Before sampling, all subjects had fasted for at least 12 h. Blood samples were collected into glass tubes without anticoagulant. Serum samples were divided into 200 µL aliquots and kept frozen at −20°C until analysis. All subjects gave written informed consent to participate in this study. All samples were provided anonymously. This study was approved by the Local Ethics Committee at the Institute of Immunology and the investigation was performed according to the ethical guidelines in Document No. 79-11/12, 11 October 2012.

### 3. Conjugation of Anti-human IgE Antibodies with Quantum Dots (Anti-IgE-QD)

The procedure is based on the technique presented in reference [31]. The surface carboxyl groups of the QDs were activated by EDC and sulfo-NHS, followed by interaction with the amino groups of anti-IgE antibodies. We used a 10-fold molar excess of antibodies over quantum dots. Twenty-five microliters of quantum dots dissolved in BB at a concentration of 8 µmol/L were mixed with 300 µL of antibody solution prepared in PBS 10 at a concentration of 1 g/L. Stock solutions of EDC and sulfo-NHS in deionized water were prepared at a concentration of 1.6 • 10^−3^ mol/L. 50 µL of each activator solution was added into the reaction mixture. The molar ratio of QDs to activator was 1∶400. The reaction was performed with constant mixing on an Intelli-Mixer RM-2 shaker (ELMI, Riga, Latvia) for 90 min at room temperature in the dark. After the reaction, the mixture was concentrated and simultaneously dialyzed against BB to remove excess activator. The antibody-labeled QDs were isolated by centrifugal filtration five times in Amicon Ultracel 100 K centrifugal filter units at 10,000 g for 15 min each time. The collected conjugate was concentrated 10 times.

Gel filtration, on columns packed with Sephadex G-100 and equilibrated with PBS 10 was performed to further purify the conjugate from free low-molecular-weight substances and unconjugated antibodies The conjugate was diluted 20 times with PBS 10 and placed in the upper part of the column, followed by separation using the same buffer solution as an eluent. All fractions exhibiting color and/or fluorescence were collected. All fluorescent fractions were tested for binding by an immunochromatographic method, and those that showed maximum fluorescence intensity upon binding to the test zone were pooled.

Fractions obtained after gel filtration were again concentrated and simultaneously dialyzed against BB by 10 centrifugations at 10,000 g for 15 minutes each time in Amicon Ultracel 100 K centrifugal filter units. After the last centrifugation, the 10-fold concentrated conjugate was transferred into a microcentrifuge tube and stored at 4°C.

### 4. Preparation of Test Strips for the Determination of Total IgE in Human Serum

A solution of monoclonal anti-human IgE antibodies, dissolved in PBS 50 to a concentration of 3 g/L, was used to prepare the test zone. A solution of goat anti-mouse IgG polyclonal antibodies, dissolved in PBS 50 to a concentration of 1 g/L, was used to prepare the control zone. Aliquots of the antibody solutions were applied to a CNPC12 nitrocellulose membrane via an IsoFlow dispenser (Imagene Technology, Hanover, PO, USA) [32], [33]. Both loading solutions were applied at volumes of 0.1 µL per millimeter of membrane width. Antibody-conjugated QDs at a QD concentration of 10 nmol/L in BB containing 7.5% BSA were applied to a fiberglass membrane at a volume of 2 µL per millimeter of membrane width. The membranes were incubated at room temperature for 20 h to dry.

The complete test structures were then assembled, and the CNPC12 nitrocellulose membrane with immobilized antibodies, the fiberglass macroporous membrane (CFCP203000) coated with the antibody-QD conjugates, a sample pad (R7L) and an adsorption pad (CFSP223000) were comprised. The multimembrane composite was cut into individual 3.5-mm-wide test strips by a guillotine cutter. These test strips were vacuum packed, with silica gel as a desiccant, in bags of laminated aluminum foil by using an FR 900 solder with a mini conveyor (Wenzhou Dingli Packing Machinery Co., China). Cutting and packing were conducted at 20–22°C in a climate-controlled room with relative air humidity not exceeding 30% [34]. Test strips were used immediately after their manufacture; additional experiments determined that their shelf life is at least 1 year.

### 5. Determination of Total IgE in Human Serum by ELISA

The total level of IgE in the human serum samples was determined via ELISA using the Total IgE HRP EIA (Dr. Fooke Laboratorien, Neuss, Germany) according to the manufacturer’s instructions. A 100 µL of the dilution buffer was added to each well containing adsorbed mouse antibodies against human IgE. A 20 µL of either the serum sample or the total IgE positive/negative control sera was then added, and the plates were incubated for 30 min at room temperature. This step was followed by further incubation with100 µL/well anti-IgE antibodies conjugated to horseradish peroxidase. The plate was washed 3 times with washing buffer between each step. TMB substrate (100 µL/well) was then added, and the plates were incubated for 15 min at room temperature in the dark until the color developed, at which point, TMB peroxidase stop solution was added (50 µL/well). The optical density at 450 nm was measured by a Zenyth 3100 microplate multimode detector from Anthos (Wals, Austria).

### 6. Determination of Total IgE in Human Serum by Immunochromatography

Samples and test strips were brought to room temperature (20–25°C) prior to analysis. A 20 µL of 5% Tween 20 was added to a 100-µL sample, and the solution was mixed. A test strip was immersed into the sample for 10 min, removed from the sample, and analyzed while wet. Fluorescence intensity in the test area was measured using a portable detector REFLEKOM-UV containing a UV light source equipped with Videotest software (Synteco-Complex, Moscow, Russia).

Calibration curves were obtained using the standard solutions, by plotting the relationship between the known IgE concentration and the fluorescence intensity in the test zone. Based on the calibration curves, IgE concentration in the samples was estimated using the four-parameter sigmoidal and exponential equations in Origin 7.5 (OriginLab, Northampton, MA, USA) software.

## Results and Discussion

### 1. Preparation and Characterization of Antibody-conjugated Quantum Dots

Anti-human IgE (clones 4F4 and ME-113) antibodies were conjugated with quantum dots by the activated ester technique [35]. The molar ratio of QDs, IgG, EDC, and sulfo-NHS was 1∶10:400∶400. We examined the fluorescent properties of the conjugates and concluded that the fluorescence of the antibody-conjugated QDs had similar intensity and peak wavelengths to that of the native QDs (data not shown). This indicates that the fluorescence of the QDs was similar before and after conjugation, and was not affected by the reactants at the concentrations employed.

We checked the differences of unconjugated and conjugated QDs by transmission electron microscopy, dynamic light scattering and immunochromatography. Transmission electron microscopy confirmed the absence of nanoparticles aggregation as well as the increase of particle sizes after the conjugation compared to unconjugated QDs. The same increase was found for data of dynamic light scattering; size values for this technique were higher due to impact of hydration shell. Immunochromatography results indicate the appearance of specific antigen-binding capacity of conjugated QDs (Fig. S1–S4).

### 2. Optimal Conditions for Immunochromatographic Analysis

The proposed method for IgE immunochromatographic assay is based on the “sandwich” immunoassay format. Anti-human IgE antibodies labeled with QDs bind to IgE contained in the sample. This complex in turn reacts with anti-human IgE antibodies applied to the working membrane.

Buffer compositions (see “Materials and methods”) were selected based on previous results obtained for the immunochromatographic test system for determination of specific human serum IgE for the diagnosis of allergy to pollen, timothy meadow, with colloidal gold [32].

Anti-human IgE antibodies, clones 4F4 and ME-113, and were immobilized on the working membrane from a solution with the concentration of 1 g/L. Upon UV irradiation of the test strip, maximum fluorescence intensity in the test zone was achieved by a combination of the clone 4F4, immobilized on a nitrocellulose membrane, and the clone ME-113 labeled with quantum dots (Table 1). All other combinations were characterized by substantially lower fluorescence intensity (at least 2.3-fold), and were excluded from further experiments.

**Table 1:** Fluorescence intensity in the test zone of an immunochromatographic test strip at different combinations of antibodies against human IgE.

	Antibodies immobilized in the test zone	
	Clone 4F4	Clone ME-113
Antibodies conjugated to QDs	Fluorescence intensity, relative units	
Clone 4F4	1.3	2.1
Clone ME-113	4.8	0.6

The QDs-conjugates of the same clones of antibodies were used at a concentration of 0.8 µmol/L (QD concentration).

A standard solution of IgE with a concentration of 200 kU/L was used as a sample.

**Figure 1:**
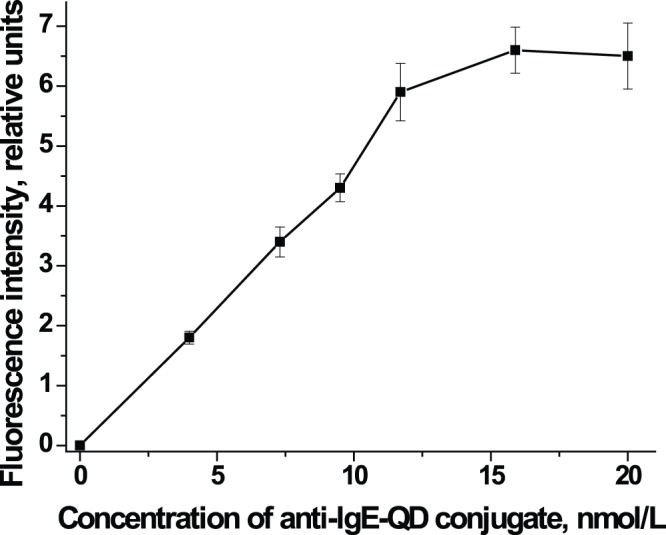
Selecting the optimal concentration of anti-IgE-QD. The figure shows the relationship between fluorescence intensity in the test zone of the immunochromatographic assay and the concentration of anti-IgE-QD conjugate. A standard solution of IgE with a concentration of 200 kU/L was used as a sample.

We then optimized the concentration of anti-IgE-QD conjugate. Changing the concentration of the conjugate from 4 to 20 nmol/L showed that the fluorescence intensity in the test zone increases with conjugate concentration and reaches a maximum at 12 nmol/L (Fig. 1). A conjugate concentration of 12 nmol/L or higher yielded high background fluorescence due to excess conjugate, which became uniformly distributed on the surface of the membrane. Thus, we determined that 10 nmol/L was the optimal concentration of the conjugate, and used this concentration for further experiments.

**Figure 2:**
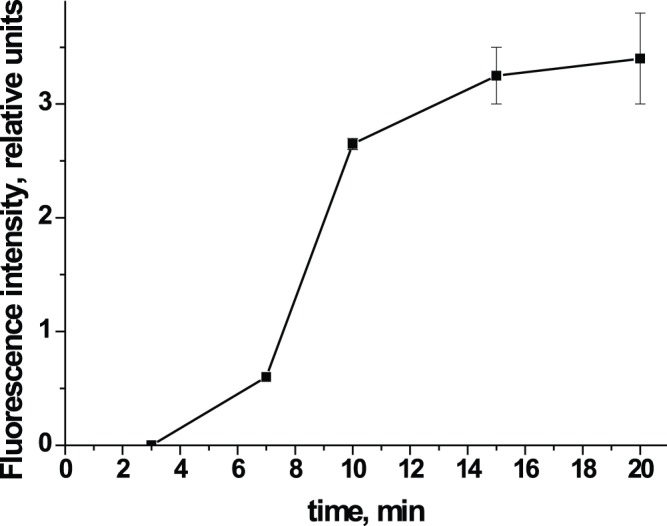
Change in fluorescence intensity with analysis time. The figure shows the relationship between the analysis time and the fluorescence intensity in the test zone of the immunochromatographic test system. A standard solution of IgE with a concentration of 200/L was used as a sample.

Next, we investigated the relationship between the fluorescence intensity and the duration of the analysis. Fig. 2 shows that the fluorescence intensity in the test zone reaches its maximum at 10 min after the start of fluid migration along the membrane. Therefore, 10 min was chosen as the optimal analysis time.

### 3. Characterization of Test System

**Figure 3:**
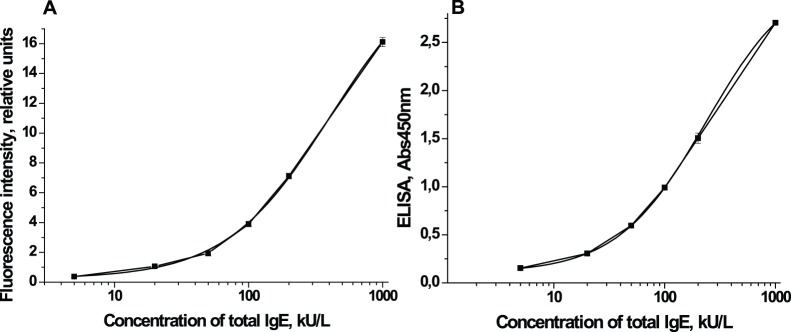
Calibration curves for the determination of total IgE in human serum. **A -** Calibration curve of the immunochromatographic assay for total IgE in human serum. The calibration curve for the determination of total IgE in human serum by using the developed immunochromatographic assay (n = 10) was obtained. **B** - Calibration curve of the ELISA for total IgE in human serum. The calibration curve for the determination of total IgE in human serum by using the Total IgE EIA (n = 8) was obtained.

Using the optimal conditions determined in Section 2, and IgE standard solutions (Dr. Fooke Laboratorien, Nessen, Germany), we constructed a calibration curve for immunochromatographic analysis using our test system (Fig. 3A). The calibration curve provides an estimate of the total IgE over a concentration range from 5 to 1000 kU/L, and was used to analyze samples of serum from healthy subjects. The standard deviations in the operating range of concentrations varied from 2.0 to 9.5% (Table 2); the average standard deviation was 5.7%.

**Table 2:** Estimation of total IgE by immunochromatographic assay and ELISA: mean (n = 8) and standard deviation.

[IgE], kU/L	Immunochromatography		ELISA	
	Mean, relative units	SD, %	Mean, OD_450_	SD, %
5	0.38	9.3	0.15	1.0
20	1.06	6.6	0.31	2.1
50	1.94	9.5	0.59	0.9
100	3.90	4.6	0.99	0.2
200	7.12	2.5	1.50	5.4
1000	16.1	2.0	2.70	0.6

To determine the accuracy of the analysis, and to confirm differences between negative and positive samples, we also analyzed samples containing low (2 and 5 kU/L) levels of total IgE and samples without IgE (0 kU/L). Ten replicates of each sample were analyzed. Table 3 shows that in the absence of IgE, fluorescence intensity in the test zone is zero. The detection limit was 2 kU/L; at this level of IgE, however, the standard deviation was 19.2% because of the low intensity of fluorescence in the test zone. Analysis of a standard solution containing 5 kU/L of total IgE showed that the standard deviation did not exceed 9.7% (Table 3), confirming the accuracy of the analysis.

**Table 3:** Estimation of total IgE by immunochromatographic assay: mean (n = 10) and standard deviation.

[IgE], kU/L	Mean, relativeunits	SD, relative units	SD, %
5	0.41	0.04	9.7
2	0.26	0.05	19.2
0	0.00	0.00	0.0

When the calibration curve for the immunochromatographic analysis was plotted in the linear coordinates, it could be described by an exponential model (Equation 1) [36].
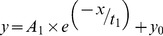
(1)


The calibration curve of ELISA (Fig. 3B) was obtained using the Total IgE HRP EIA kit and the standard solutions of IgE from Dr. Fooke Laboratorien (Nessen, Germany). According to the manufacturer’s instructions, the calibration curve was plotted on a semi-logarithmic coordinate system and described by a four-parameter sigmoidal function (Equation 2). The same was also done for the immunochromatography assay.
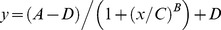
(2)


**Figure 4:**
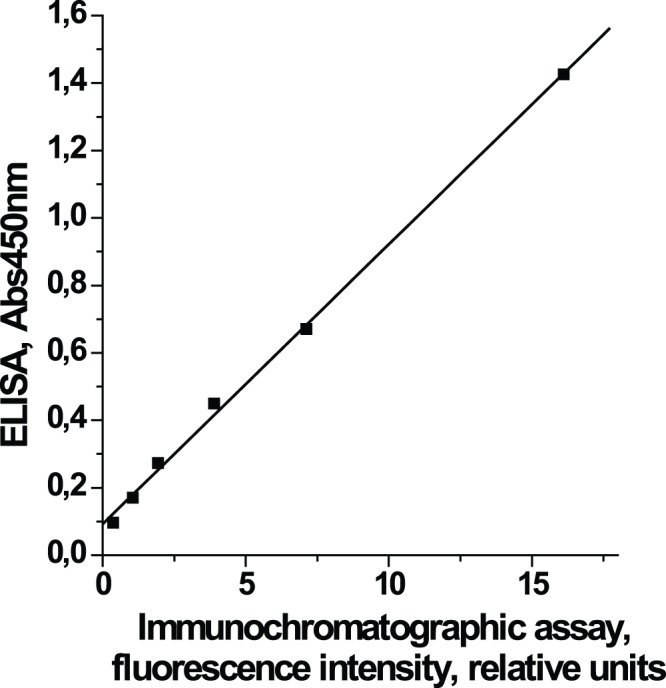
Correlation between the results of ELISA and immunochromatographic assay. Estimation of human IgE in the standard solutions by the QD-based immunochromatographic assay is strongly correlated with the results obtained using a commercial ELISA kit (R^2^ = 0.9989).

Fig. 4 shows that measurements of IgE standards by immunochromatography are well correlated with those obtained by ELISA (R^2^ = 0.9989). Thus the immunochromatographic data can be analyzed using the same model as data of ELISA.

The given four-parameter sigmoidal model was used for further work.

**Figure 5:**
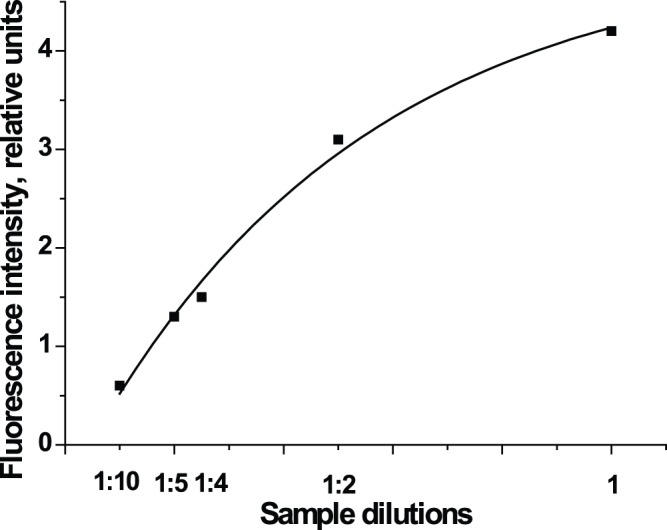
Relationship between sample dilution and fluorescence intensity in the test zone. A sample containing 100/L of total IgE was used at various dilutions. R^2^ = 0.9687.

Next, we investigated the influence of the sample matrix on the immunochromatography results. IgE samples were prepared from a serum sample containing 100 kU/L of total IgE, which was used undiluted and also diluted by 2- to 10-fold with PBS 50. Fig. 5 shows that serum dilution did not affect the relationship between the analytical signal and the concentration of total IgE, thus validating the equation of the calibration curve. These results demonstrate the ability of the immunochromatography assay to accurately determine total IgE levels in human serum even at a sample dilution of up to 10-fold.

### 4. Advantages of the Developed Immunochromatographic Assay in Comparison with Existing Methods of IgE Detection Used Nanocrystals

As indicated above, there are several publications describing application of nanocrystals for the immunodetection of human IgE by using such techniques as ELISA [28] and phosphorescent immunoassay [29]. Yao and coworkers [28] described the application of nonfluorescent nanocrystals for for IgE determination. Nanocrystals were used as a carrier for antibodies against human IgE and after cation exchange the content of bound Zn^2+^ is measured. The limit of IgE detection was 5 pg/mL, but this assay necessitates about 2 days for providing. Liu and coworkers [29] described the application of CdSe/CdS quantum dots modified with cysteine in phosphorescent immunoassay. These nanocrystals were conjugated with antibody against human IgE, and the assay duration is about 20 hours.

However, the both earlier described methods require long time for the analysis (20 hours or more), and due to this are less suitable for medical practice as compared with the developed immunochromatographic assay (the analysis time is 10 min).

### 5. Testing the Immunochromatographic Assay in Serum Samples from Patients

**Figure 6:**
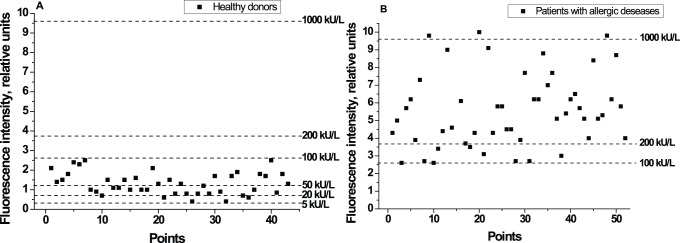
Eatimation of total IgE in human serum for healthy donors and patients with allergic diseases. The fluorescence intensity was determined in test area in accordance with total IgE content in probe. **A -** Estimation of total IgE for 43 healthy donors. **B -** Estimation of total IgE for 52 patients with allergic diseases.



We used 95 serum samples obtained from adult peoples to test the developed method. All samples were characterized by ELISA using the Total IgE HRP EIA kit (Dr. Fooke Laboratorien, Nessen, Germany). The 95 sera included 43 samples from healthy donors and 52 samples from the patients with allergic diseases. We have compared data for these two groups for further evaluation of the applicability of the developed immunochromatographic method. The variations in the fluorescent signal for serum samples from healthy donors and from patients with allergic diseases are shown on Fig. 6. Based on the fluorescence intensity we established total IgE concentration in each serum sample. Fig. 6A and 6B indicate that the developed assay demonstrates significant differences of detected signals between healthy people and patients with allergic diseases. When testing QD-based immunochromatographic method, the content of total IgE in each sample was determined from the corresponding calibration curves and by applying sigmoidal (Equation 3) and exponential (Equation 4) approximations:

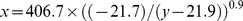
(3)

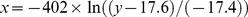
(4)


The approximations in Equations 3 and 4 were both used to estimate the total IgE from immunochromatography of five randomly selected serum samples. The data (Table 4) indicate that both quantification methods are appropriate. These data also correlated with the results obtained by ELISA, thus any of the equations described above could be used. The percentage of consistency between immunochromatography and ELISA, given by the Equation 5, ranged from 79 to 92%. Values of C_sigmoidal_/C_exp_ ranged from 0.97 to 1.09, thus indicating good correlation between the sigmoidal and exponential functions for interpreting the immunochromatographic results.

**Table 4:** Comparison of the sigmoidal and exponential equations for determining the concentration of total IgE from the calibration curve.

		Immunochromatographic analysis		
		Percentage of consistency for total IgE		C_sigmoidal_/C_exp_
Sample	[IgE] (kU/L)	Sigmoidal equation	Exponential equation	
1	15	87	81	1.07
2	42	86	79	1.09
3	330	81	80	1.01
4	631	83	86	0.97
5	1000	92	91	1.01

The reference value of total IgE (second column from the left) is given by the results from a commercial ELISA test kit.



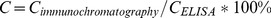
(5)


**Figure 7:**
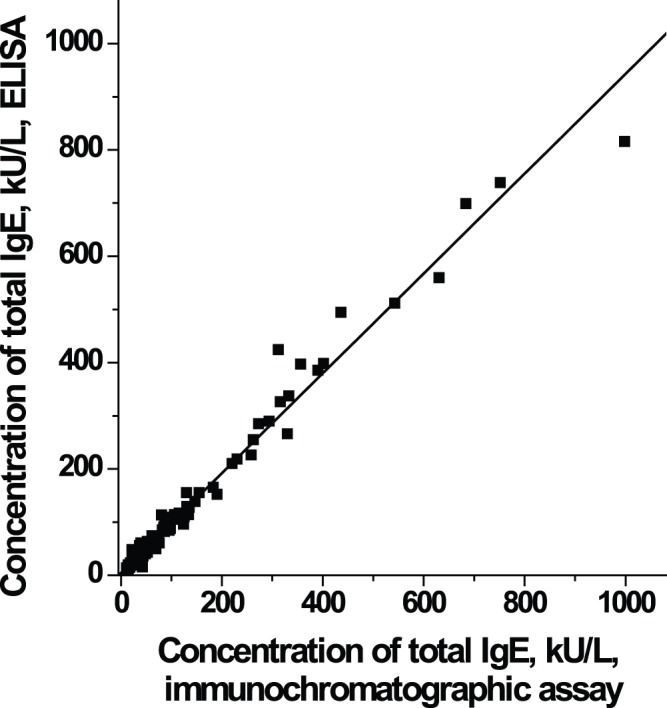
Correlation between ELISA and immunochromatographic assay results for samples from 95 human subjects. The immunochromatographic assay is able to estimate total IgE in the serum of human subjects, with good correlation kit (R^2^ = 0.9884) to the results of a commercial ELISA.

Because nearly identical results were obtained by both methods of calculation, we chose the sigmoidal equation because it unifies processing of data acquired by both ELISA and immunochromatography. This equation was used to calculate the IgE content in 95 serum samples. As Fig. 7 shows, the results obtained by ELISA and immunochromatography correlated well, with an R^2^ of 0.9884.

## Conclusions

We have developed a quantum-dot-based immunochromatographic analysis method for the rapid quantitative determination of total IgE in human serum over a wide range of concentrations, 5–1000 kU/L and the coefficient of variation was less than 10%. Fluorescence of the bound label was measured using a portable handheld detector. The obtained results were well correlated (R^2^ = 0.9884) with the data obtained by a commercial ELISA kit.

## Supporting Information

Figure S1 **Electron micrographs of unconjugated quantum dots (A) and quantum dots conjugated with anti-human IgE antibodies (B).**
(EPS)

Figure S2 **Size distribution of unconjugated quantum dots (A, mean size 8,9±0,1 nm) and quantum dots conjugated with anti-human IgE antibodies (B, mean size 13,8±0,2 nm) determined by transmission electron microscopy.**
(EPS)

Figure S3 **Size distribution of unconjugated quantum dots (A, mean diameter 24.2±1.2 nm) and quantum dots conjugated with anti-human IgE antibodies (B, mean diameter 29.8±1.0 nm) determined by dynamic light scattering.**
(EPS)

Figure S4 **Images of the test strips indicating specific conjugate binding.** A – positive reaction in test and control lines owing to the interaction of anti-human IgE antibodies labeled by quantum dots with antibodies immobilized on the membrane. B – unconjugated quantum dots do not interact with the same antibodies immobilized.
(EPS)

Methods S1 **Transmission electron microscopy; Dynamic light scattering; Immunochromatographic testing of conjugates binding.**
(DOC)
