# Supplementary material for: Quantum-Dot-Based Immunochromatographic Assay for Total IgE in Human Serum
Source: PLoS One. 2013 Oct 30;8(10):e77485. doi: 10.1371/journal.pone.0077485 (PMC3813722; doi:10.1371/journal.pone.0077485)
Supplement: Methods S1 — Transmission electron microscopy; Dynamic light scattering; Immunochromatographic testing of conjugates binding. (DOC) [file pone.0077485.s005.doc]

**Methods S1**

1. **Transmission electron microscopy**

Preparations of native and conjugated quantum dots were applied to 300-mesh grids (Pelco International, Redding, CA, USA) coated with a support film of polyvinyl formal deposited from chloroform. The images were obtained with a JEM CX-100 electron microscope (JEOL Ltd., Tokyo, Japan) operating at 80 kV. The digital images were analyzed with the Image Tool program (University of Texas Health Science Center, San Antonio, TX, USA).

1. **Dynamic light scattering**

Solutions of native and conjugated quantum dots (10 nmol/L) were dropped into the cylindrical glass cuvette with the inner diameter 6.3 mm and preincubated for 5 minutes (the temperature was 26 ° C). The data were obtained using a Photocor equipment for dynamic light scattering (Photocor Instruments Inc., College Park, MD, USA), with a He-Ne laser (wavelength 632.8 nm, power 10 mW, Coherent, USA). The measurements were provided at an angle of 90 degrees by the correlator Photocor-FC.

1. **Immunochromatographic testing of conjugates binding**

The solutions of unconjugated quantum dots and quantum dots conjugated with anti-human IgE antibodies (4 nmol/L) were dropped onto the glass-fiber membranes and dried. The obtained pads were used for preparation of test strips as described in Section 2.4 of the manuscript. Serum sample containing 100 kU/L of total IgE and test strips were brought to room temperature (20–25 °C) prior to analysis. A 20 μL of 5% Tween 20 was added to a 100-μL sample, and the solution was mixed. A test strip was immersed into the sample for 10 min, removed from the sample, and analyzed while wet. Fluorescence was detected using a portable detector REFLEKOM-UV containing a UV light source.
